# Supplementary material for: Rotavirus A Inoculation and Oral Vitamin A Supplementation of Vitamin A Deficient Pregnant Sows Enhances Maternal Adaptive Immunity and Passive Protection of Piglets against Virulent Rotavirus A
Source: Viruses. 2022 Oct 26;14(11):2354. doi: 10.3390/v14112354 (PMC9697517; doi:10.3390/v14112354)
Supplement: Supplementary file 1 [file viruses-14-02354-s001.zip › S1 data/B cell manuscript supplementary data 10-7-22.pdf]

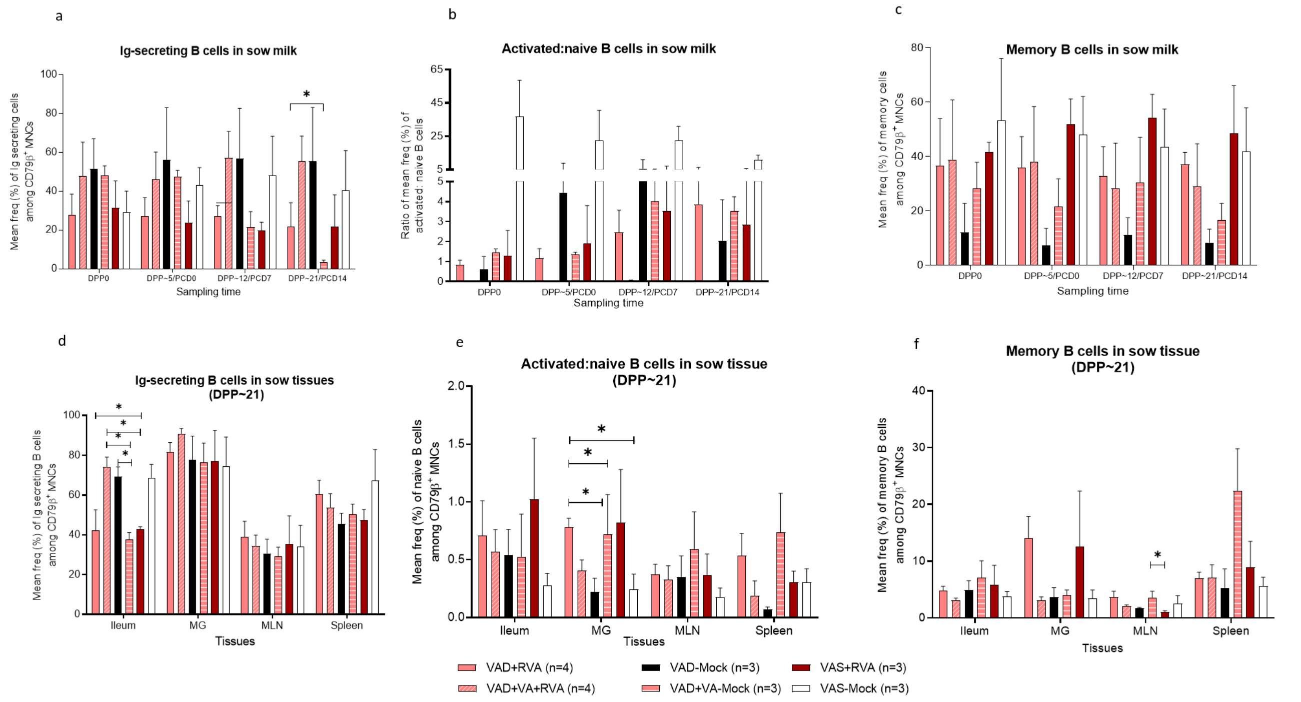

Figure S1. Mean frequencies (%) of Ig secreting B cells (a,d), ratio of activated vs. naïve B cells (b,e), memory B cells (c,f) cell subpopulations among B lymphocytes (CD79β+) in milk and tissues respectively of RVA (OSU)/mock inoculated sows fed VAS and VAD(±VA) diets during gestation and postpartum. * $p < 0.05$.
